# Does time-lapse imaging have favorable results for embryo incubation and selection compared with conventional methods in clinical in vitro fertilization? A meta-analysis and systematic review of randomized controlled trials

**DOI:** 10.1371/journal.pone.0178720

**Published:** 2017-06-01

**Authors:** Minghao Chen, Shiyou Wei, Junyan Hu, Jing Yuan, Fenghua Liu

**Affiliations:** 1 Reproductive Center, Guangdong Women and Children Hospital, Guangzhou, China; 2 Department of Thoracic Surgery, West China Hospital, Sichuan University, Chengdu, China; 3 Department of Emergency, The Third Affiliated Hospital of Guangzhou Medical University, Guangzhou, China; 4 School of Traditional Chinese Medicine, Jinan University, Guangzhou, China; Universite Blaise Pascal, FRANCE

## Abstract

**Objective:**

The present study aimed to undertake a review of available evidence assessing whether time-lapse imaging (TLI) has favorable outcomes for embryo incubation and selection compared with conventional methods in clinical in vitro fertilization (IVF).

**Methods:**

Using PubMed, EMBASE, Cochrane library and ClinicalTrial.gov up to February 2017 to search for randomized controlled trials (RCTs) comparing TLI versus conventional methods. Both studies randomized women and oocytes were included. For studies randomized women, the primary outcomes were live birth and ongoing pregnancy, the secondary outcomes were clinical pregnancy and miscarriage; for studies randomized oocytes, the primary outcome was blastocyst rate, the secondary outcome was good quality embryo on Day 2/3. Subgroup analysis was conducted based on different incubation and embryo selection between groups.

**Results:**

Ten RCTs were included, four randomized oocytes and six randomized women. For oocyte-based review, the pool-analysis observed no significant difference between TLI group and control group for blastocyst rate [relative risk (RR) 1.08, 95% CI 0.94–1.25, I2 = 0%, two studies, including 1154 embryos]. The quality of evidence was moderate for all outcomes in oocyte-based review. For woman-based review, only one study provided live birth rate (RR 1,23, 95% CI 1.06–1.44,I2 N/A, one study, including 842 women), the pooled result showed no significant difference in ongoing pregnancy rate (RR 1.04, 95% CI 0.80–1.36, I2 = 59%, four studies, including 1403 women) between two groups. The quality of the evidence was low or very low for all outcomes in woman-based review.

**Conclusions:**

Currently there is insufficient evidence to support that TLI is superior to conventional methods for human embryo incubation and selection. In consideration of the limitations and flaws of included studies, more well designed RCTs are still in need to comprehensively evaluate the effectiveness of clinical TLI use.

## Introduction

Cultivation of fertilized oocytes and the subsequent selection of embryos is a vital step in assisted reproductive technology. Since the inception of clinical in vitro fertilization (IVF), most laboratories have routinely selected embryos on the basis of morphological characteristics. This method requires moving embryos outside the controlled environment of the incubator for microscopic examination. However, owing to the potentially deleterious effect of exposure embryos to undesirable changes in culture environment when evaluated, inspection of embryo morphology is limited to snapshots at a few discrete time points (usually once a day), reducing the amount of information that could be obtained and important developmental events might be missed. Conventional embryo selection methods are commonly associated with relatively low clinical pregnancy rate of approximately 30% per transfer [1]. Nowadays, promoting of single embryo transfer (SET) and minimizing the rate of multi-pregnancy put higher demand on the efficiency of identifying the most viable embryo for transfer, and many new technologies aiming at improving embryo selection have be developed[2, 3].

Time-lapse imaging (TLI) is an emerging technology, clinical available TLI systems allow continuous observation of embryo development without removal from controlled and stable incubator condition. TLI systems may improve outcomes of IVF cycles over conventional methods due to several potential advantages. First, decreased frequency of handling and exposure of embryos to suboptimal conditions eliminates the risks of stress from temperature changes, high oxygen exposures and pH changes in the culture medium and thus provides improved culture conditions. Second, by serial imaging, more information on embryo development is obtained. TLI allows embryologists to assess the quality of embryos by tracking the timing of events and length of different intervals in embryo development (also refers to as morphokinetic monitoring), adds another dimension to embryo selecting and grading. Until now, a number of studies have been conducted regarding whether morphokinetic variables are correlated with important outcomes such as blastocyst formation[4–8], implantation potential[9, 10], pregnancy potential[11, 12] and even aneuploidy status[13–16]. Several predictive algorithms based on morphokinetic parameters and time-lapse evaluation have been proposed[8, 12, 17–19].

As stated, the potential benefit of clinical TLI may be due to improved incubation or improved embryo selection or both, the relative contribution of each variable needs to be acknowledged. Available RCTs detecting the effectiveness of TLI so far can be divided in to 3 types: studies comparing TLI incubation to conventional incubation, studies assessing the overall impact of TLI to conventional methods and studies focusing on the potential effect of TLI morphokinetic monitoring and embryo selection. A few meta-analyses and systematic reviews evaluating TLI have been published including a Cochrane meta-analysis[20–23]. However, these reviews focused on either the mixed effect of TLI or the effect of TLI selection alone.

On the other hand, distinct light exposure, heat due to motion, friction of moving parts, presence of magnetic fields, sheer stress of moving culture dishes and lubricants related to TLI systems may bring harmful effect on embryo quality. Considering the conflicting results of studies comparing TLI incubation to conventional incubation, the safety of TLI systems needs to reconfirm[24–28]. Besides, increased embryo assessment time (301.2 seconds vs. 137.6 seconds up to Day 3)[28], considerable increased expense of equipment and consumable materials and extra space needed on the laboratory are the disadvantages of TLI which make assisted reproduction even less accessible. As such, it is urgent to perform a meta-analysis and systematic review on larger samples to comprehensively compare the effectiveness of TLI to conventional methods and provide a more precise estimation of TLI clinical use.

## Methods

### Eligibility criteria

Randomized controlled trials (RCTs) that compared TLI with conventional methods were considered eligible. We only included true RCTs, quasi- or pseudo- randomized trials were excluded. Both studies that randomized oocytes and studies that randomized women were included, however, these studies were analyzed separately as a woman-based review and an oocyte-based review.

### Information source

A systematic search of PubMed, EMBASE, Cochrane library and ClinicalTrials.gov up to February 2017 was conducted. The following terms were used, adjusting for each database as necessary: (time-lapse OR “time lapse” OR EmbryoScope OR EEVA OR “Primo Vision” OR “live cell imaging” OR “closed system”) AND (“in vitro fertilization” OR IVF OR “introcytoplasmic sperm injection” OR ICSI OR “assisted reproductive technology” OR “assisted reproduction” OR ART OR embryo OR blastocyst). Limitation categorical terms were used: human, clinical trials. Additionally, the reference lists of included studies and related reviews were also manually-searched to identify potentially relevant studies. There was no limitation regarding the language, publication date or publication status.

### Study selection

Two reviewers (CMH and WSY) independently examined the searching results of possible relevant trials and full-texts were obtained when necessary. Disagreements were solved by group discussion. We contacted the original authors by e-mail when necessary.

### Data collection

All authors independently extracted data from each included study using a standardized data-extraction form. If necessary, we contacted the authors to retrieve missing data. In the case of duplicate publications with accumulating numbers of patients or increased lengths of follow-up, we included the most recent or complete data for analysis.

### Date items

Related information was sought for the following variables: first author’s name, year of publication, country, incubation and embryo selection of two groups (TLI group and control group), period of enrollment, type of TLI system, number of women included, number of oocytes included, inclusion and exclusion criteria for women, fertilization method, number of embryos transferred, day for transfer, fresh or frozen cycles, oocyte source, culture parameters, sample size calculation, implantation rate, embryo assessment time usage, economical burden and extra place needed in laboratory. For woman-based review, the primary outcomes were ongoing pregnancy and live birth, the secondary outcomes were clinical pregnancy and miscarriage. For oocyte-based review, the primary outcome was blastocyst formation and the secondary outcome was good quality embryo on Day 2/3. For the definition of good quality embryos, any classification criteria was accepted.

### Assessment of the risk of bias and quality of evidence

Two reviewers (CMH and WSY) independently assessed the quality of included RCTs according to the Cochrane Collaboration’s Handbook[29]. The risk of selection bias (random sequence generation and allocation concealment), performance and detection bias (blinding of participants and blinding of outcome assessors), attrition bias (incomplete outcome data), reporting bias (selective outcome reporting), and other potential bias (e.g., co-interventions and age of participants) were evaluated and low, high or unclear risk of bias was classified for each included RCT.

To minimize the potential impact of reporting bias, we attempted to perform a comprehensive search for eligible studies, avoiding language restriction, broadening eligibility to conference proceedings and grey literatures, and maintaining alertness for duplication of data.

The overall quality of the body of evidence was assessed in a structured way using the GRADE criteria[30]. This accounted for study limitations (i.e., risk of bias), consistency of effect, imprecision, indirectness, and suspicion of publication bias.

### Summary measures and statistical analysis

Relative risk (RR) with corresponding 95% confidence intervals (CIs) was used to assess the overall effects for discontinuous data.

All analyses were conducted with Review Manager Version 5.3 (Cochrane Collaboration, Software Update, Oxford, United Kingdom, 2014) in a random-effect model since the interventions varied among included studies. Statistical heterogeneity among the study results was examined by the P value and I^2^ statistic. A I^2^ value of ≥50% was considered to indicate substantial heterogeneity. Any observed heterogeneity was taken into account in the interpretation of the estimates. Subgroup analysis was conducted based on different incubation and embryo selection between groups: TLI incubation + conventional selection vs. conventional incubation and selection, TLI incubation and selection vs. conventional incubation and selection and TLI incubation and selection vs. TLI incubation + conventional selection.

## Results

### Study selection

**Fig 1:**
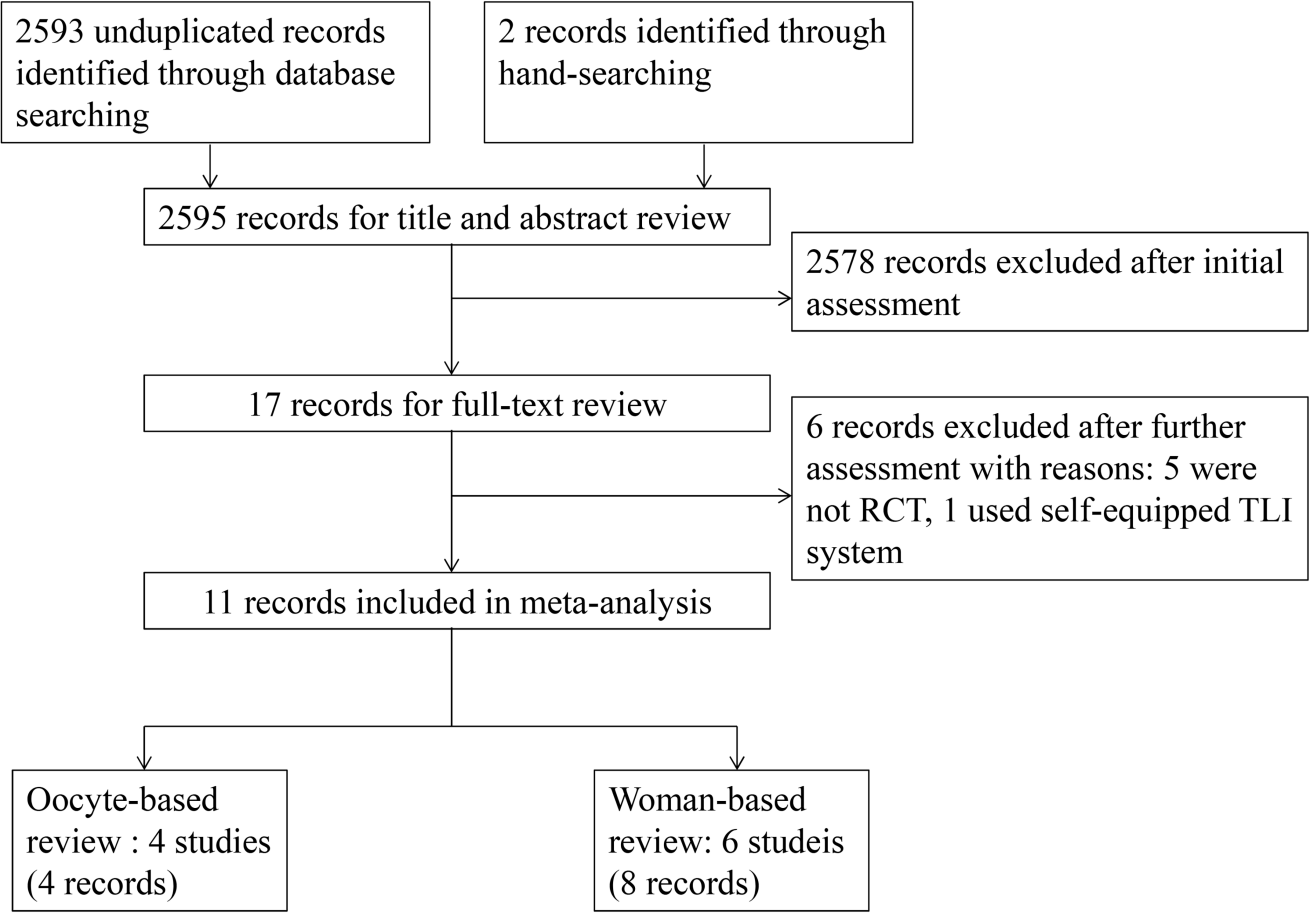
Flow chart for the systematic review of RCTs comparing TLI with conventional method.

2593 no duplicate records were identified in the initial electronic search, and two records were retrieved by manual-search of the reference list of relevant studies. After reading titles and abstracts, 2578 records clearly did not meet the eligible criteria, and 17 records were selected to undergo further full-text review. Among these studies, five were excluded because they were not randomized[12, 31–34]; one used a self-equipped TLI system and we excluded this study because the culture condition in the self-equipped system was hardly as stable as in a commercial instrument[35]. At the end, ten studies (eleven records) assessing the efficiency of TLI versus conventional methods in women undergoing IVF met our inclusion criteria and were included in our analysis. Four studies randomized oocytes and six randomized women. The flow chart of the studies included in the systematic review and meta-analysis is shown in Fig 1. Six studies compared TLI incubation and conventional embryo selection to conventional incubation and embryo selection (four randomized oocytes and two randomized women)[24–28], three studies compared TLI incubation and embryo selection to conventional incubation and embryo selection (all three randomized women)[36–40] and only one study compared TLI incubation and embryo selection to TLI incubation and conventional embryo selection (this study randomized women)[41]. Regarding the difference between the number of records and studies, two studies had two records each[24, 37, 38, 40]; two studies, one randomized women and one randomized oocytes, were reported in the same article[28].

### Characteristics of included studies

Main characteristics of included studies are shown in Table 1.

**Table 1:** Characteristics of included randomized controlled studies.

Study/country/enrollment/incubation and embryo selection of two groups	Inclusion criteria/Exclusion criteria	Implantation rate/Age (year) (TLI group vs. control group)	ART, embryo transfer and oocyte source	TLI system and culture characteristics	Control group culture characteristics^a^	Sample size calculation
*Studies randomized oocytes*						
Nakahara 2010/Japan/April 2007 to September 2008/ TLI incubation + conventional selection vs. conventional incubation and selection	NR/NR	NA/average 36.8 years for all included patients.	ICSI; 1–2 embryos for transfer on Day 3; fresh or vitrified embryo transfer not reported; autologous or donation cycles not reported.	SANYO In Vitro Live Cell Imaging Incubation System; 37°C,5% CO_2_ and 95% air atmosphere; embryos were not removed from the incubator for assessment; images were obtained every 15 min.	37°C,5% CO_2_ and 95% air atmosphere.	No sample size calculation.
Cruz 2011/Spain/study duration not reported/ TLI incubation + conventional selection vs. conventional incubation and selection	Patients aged 32–45 years old./Patients with pathologies like endometriosis, hydrosalpynx, obesity (BMI>30), uterine pathology, recurrent pregnancy loss, or age >45 years old.	NA/not reported	Both ICSI and IVF; included both Day 3 and Day 5 embryo transfer; number of transferred embryos not reported; included only donation cycles.	EmbryoScope; 37°C, 6% CO_2_, pH 7.2–7.4; embryos were removed from the incubator for assessment; images were obtained every 20 min.	37°C, 6% CO_2_, pH 7.2–7.4.	Based on blastocyst rate.
Kirkegaard 2012/Denmark/June 2010 to April 2011/ TLI incubation + conventional selection vs. conventional incubation and selection	Second or third treatment cycle with a normal fertilization rate (≥50%) and embryo development in the first cycle, age <38 years, ≥8 oocytes retrieved./NR	NA/32.2±3.3 years for all included patients.	Both ICSI and IVF; 1–2 fresh blastocysts for transfer on Day 5; included autologous cycles only.	EmbryoScope; 37°C, 6% CO_2_, 20% O_2_; embryos were removed from the incubator for assessment; time interval between image acquisition not reported.	37°C, 6% CO_2_, 20% O_2_;.	Based on 4-cell proportion among inseminated oocytes.
Wu 2016 (Part B)/USA/December 2014 to March 2015/ TLI incubation + conventional selection vs. conventional incubation and selection	NR/NR	NA/27.8±1.4 years for all included patients.	ICSI; all embryo transfer were carried out on Day 3; number of transferred embryos not reported; fresh or vitrified embryos for transfer not reported; included donation cycles only.	EmbryoScope; 37°C,5% CO_2_, 5% O_2_ and 90% N_2_ atmosphere; embryos were not removed from the incubator for assessment; images were obtained every 10 min.	37°C, 5% CO_2_ and 90% N_2_.	No sample size calculation.
*Studies randomized women*						
Park 2015/Sweden/ May 2010 to February 2014/ TLI incubation + conventional selection vs. conventional incubation and selection	Female patient ≤40 years undergoing their first IVF cycle and at least 1 oocyte was retrieved./Patients undergoing egg donation.	27.9% vs. 31.6% (P = 0.32)/31.8±4.3 vs. 31.8±4.1	ICSI; 1–2 fresh embryos for transfer on Day 2 (3.3% patients received 2 embryos) for both groups; included autologous oocytes only.	EmbryoScope; 37°C, 6% CO_2_ and atmospheric O_2_ concentration; embryos were not removed from the incubator for assessment; images were obtained every 20 min.	37°C, 6% CO_2_ and atmospheric O_2_ concentration.	Based on good quality embryo on Day 2 rate.
Wu 2016 (Part A)/USA/ December 2014 to March 2015/ TLI incubation + conventional selection vs. conventional incubation and selection	Willing to participate (all patients involved were poor prognosis patients)./Patients with no mature oocytes or no fertilization, transfer on D2, IVF cycles, IVF for embryo banking.	9.7% vs. 11.5% (P>1.0)/38.8±1.0 vs. 40.4±1.8	ICSI; all transferrable embryos obtained in a cycle were transferred; fresh embryo transfer on Day 3 for both groups; included autologous oocytes only.	EmbryoScope; 37°C, 5% CO_2_, 5% O_2_ and 90% N_2_; embryos were not removed from the incubator for assessment; images were obtained every 10 min.	37°C, 5% CO_2_ and 90% N_2_.	No sample size calculation.
Kahraman 2013/Turkey/ December 2011 to June 2012/ TLI incubation and selection vs. conventional incubation and selection	Patients undergoing their first or second treatment cycle, with no recurrent spontaneous miscarriages, age <35years, BMI <28 Kg/m^2^, and ≥8 ooocytes retrieved./Patients with severe endometriosis, polycystic ovary syndrome, hydrosalpynx, uterine pathology, or severe male factor and very severe morphological sperm defects.	NR/28.5±3.32 vs. 28.5±3.72	ICSI; single Day 5 blastocyst transfer for both groups; included both fresh and vitrified transfers; included autologous oocytes only.	EmbryoScope; 37°C, 5% O_2_, 6% CO_2_; embryos were not removed from the incubator for assessment; images were obtained every 20 min.	37°C, 5% O_2_, 6% CO_2_.	Not reported.
Insua 2015/ Spain/February 2012 to July 2013/ TLI incubation and selection vs. conventional incubation and selection	Patients aged 20–38 years old, 1st/2nd ICSI cycle, BMI 18–25 Kg/m^2^./Severe male factor, hydrosalpinx, uterine diseases, endocrinopathies, recurrent pregnancy loss, endometriosis, or patients receiving concomitant medication as a treatment that might interfere with the results of the study.	44.9% vs. 37.1% (P = 0.02) /30.4±5.5 vs. 30.0±5.5	ICSI; 1–2 embryos for transfer on Day 3/5 (74% were Day 3 transfers); included both fresh and vitrified embryo transfers; included both donation and autologous cycles (48% were donation cycles).	EmbryoScope; 37°C, 5.5% CO_2_, atmospheric O_2_; embryos were not removed from the incubator for assessment; images were obtained every 15–20 min.	37°C, 5.5% CO_2_, atmospheric O_2_.	Based on clinical pregnancy rate.
Matyas 2015/Hungary/ ongoing (since 2013)/ TLI incubation and selection vs. conventional incubation and selection	Patients <36 years with normal ovarian reserve, underwent 1st/2nd cycle, had at least 3 good morphological embryos on day 3 and accepted SET./NR	NR/NR	IVF; single Day 5 blastocyst transfer for both groups; fresh or vitrified embryos for transfer not reported; included autologous cycles only.	PrimoVision; culture characteristics not reported.	Same incubator with TLI group.	Not reported.
Goodman 2016/USA/March 2014 to May 2015/ TLI incubation and selection vs. TLI incubation + conventional selection	Patients aged 18–34 years, accepted fresh cycles, had at least 4 normal fertilized zygotes./Patients had plans to undergo preimplantation genetic testing or underwent IVF for fertility preservation;	51.0% vs. 45.2% (P = 0.21)/33.6±4.0 vs. 33.3±3.9	ICSI for matured oocytes and coincubation with sperm for immature oocytes; 1–3 embryos for transfer on Day 3/5 (more than 75% were Day 5 transfers); included autologous cycles only.	EmbryoScope; 37°C, 6% CO_2_, 5.5% O_2_; embryos were not removed from the incubator for assessment; images were obtained every 10 min.	Same incubator with TLI group; embryos were not removed from the incubator for assessment	Based on clinical pregnancy rate.

a All embryos incubated in conventional incubators were removed from incubator for assessment.

ART, assisted reproductive technology; TLI, time-lapse imaging; ICSI, intracytoplasmic sperm injection.

NR, not reported; NA, not available.

For oocyte-based review, three studies used EmbryoScope (Vitrolife) as the incubation system for embryos randomized to TLI group[24, 25, 28], SANYO in Vitro Live Cell Imaging Incubation System (Sanyo) for the other one study[26]. The culture parameters for both groups were the same in all studies. Two studies randomized donated oocytes[24, 28], one study included autologous cycles only[25], and the oocyte resource was not reported in one study[26]. In two studies, embryos of both TLI and control groups were removed from the incubators for morphological assessment at the same time points[24, 25], however, in the other two studies embryos selected to TLI group were continuously incubated without interruption.

For woman-based review, five studies used EmbryoScope for TLI incubation and one used PrimoVision (Vitrolife)[38, 40]. Culture parameters of TLI group were set the same as control group in all six studies. Except one study included both autologous and donor cycles (nearly half of the patients were donor recipients)[37, 39], all studies included autologous cycles only. Two studies transferred single blastocyst on Day 5 for both groups[36, 38, 40], one study transferred 1–2 fresh embryos on Day 2 for each patient[27], one study transferred 1–3 embryos on Day 3 or Day 5 (more than 75% were Day 5 transfers)[41], the biggest study transferred 1–2 embryos in miscellaneous stages of development[37, 39] and all transferrable embryos were transferred in the smallest study that involved poor prognosis patients only[28]. Four studies reported implantation rate, there were significantly more embryos got implantation in TLI group compared to control group in one study[37, 39], but no significant difference in implantation rate was found in the other three studies[27, 28, 41]. None of the embryos from TLI group were removed out for assessment during culture in all six studies.

Only one study provided data on the time of embryo assessment and found that TLI doubled the time up to Day 3 comparing to conventional assessment[28]. Data of extra expense or extra place in laboratory was not reported in any of the included studies.

### Risk of bias in the included studies

Risk of bias judgment of all included studies is shown in Table 2.

**Table 2:** Risk of bias of included studies.

		Selection bias		Performance and detection bias		Attrition bias		Reporting bias		Other potential bias	
	study	risk	explanation	risk	explanation	risk	explanation	risk	explanation	risk	explanation
*Oocyte-based review*	Nakahara 2010	U	Method of random allocation was not described.	H	Laboratory technicians were not blinded.	L	No loss of follow-up.	L	All investigated outcomes were published.	L	None.
	Cruz 2011	U	Method of random allocation was not described.	U	Blinding unclear.	L	No loss of follow-up.	L	All investigated outcomes were published.	L	None.
	Kirkegaard 2012	L	Block randomization using random number from sealed envelopes.	L	Laboratory technicians were blinded.	H	Large proportion of oocytes dropped out after randomization.	L	All investigated outcomes were published.	L	None.
	Wu 2016 (Part B)	U	Method of random allocation was not described.	H	Laboratory technicians were not blinded.	L	No loss of follow-up.	H	Did nor report pregnancy or implantation.	L	None.
*Woman-based review*	Park 2015	L	A web based randomization programme was used.	H	Laboratory technicians were not blinded.	L	Very small proportion of women dropped out.	L	All investigated outcomes were published.	L	None.
	Wu 2016 (Part A)	L	Computer randomization was used.	H	Laboratory technicians were not blinded.	H	Large proportion of women dropped out after randomization.	L	All investigated outcomes were published.	L	None.
	Kahraman 2013	L	A computer generated list was used.	H	Laboratory technicians were not blinded.	L	Small proportion of women dropped out and reasons were clearly stated.	L	All investigated outcomes were published.	L	None.
	Insua 2015	H	Patient were able to request the intervention.	H	Patients were not blinded.	H	Loss of follow up was not balanced.	H	The authors did not report clinical pregnancy and the primary outcome (ongoing pregnancy) was not assessed as reported in the trial register	H	IVI was a part owner of and had a long-standing financial interest with Fertilitech which only recently was sold. Fertilitech is the firm that manufactures EmbryoScope
	Matyas 2015	H	Paired randomization by two envelopes, researchers were awared of allocation before enrollment.	H	Laboratory technicians were not blinded.	H	Large proportion of women (20/140) dropped out.	L	All investigated outcomes were published.	L	None.
	Goodman 2016	L	A computer-generated random number sequence was used.	H	Laboratory technicians were not blinded.	H	Large proportion of women (52/287) dropped out.	L	All investigated outcomes were published.	L	None.

L, low risk of bias; H, high risk of bias; U, unclear risk of bias.

Except one study (categorized to oocyte-based review)[24], every study deemed to be at high risk of bias in at least one domain. For three studies, there was no sufficient information retrieved from publications or from contacting the primary authors to completely assess the risk of bias[24, 26, 28]. Two studies were at high risk of selection bias related to sequence generation, in one study some patients were able to request the intervention[37, 39], in another study researchers were awared of allocation before enrollment[38, 40]. It was impossible for laboratory technicians to be blind to embryo assessment and selection unless embryos assigned to TLI group were removed out from incubator for assessment, so all studies that did not remove embryos out of TLI systems for assessment were deemed to be at high risk of performance and detection bias. Five studies were at high risk of attrition bias because large proportion of women/oocytes were dropped out after randomization[25, 28, 38–41]. One study was at high risk of reporting bias for not publishing all outcomes it set out to investigate[37, 39], the same study was also deemed to be at high risk of other bias because the IVF center was a part owner of and had a long-standing financial interest with Fertilitech which only recently was sold, Fertilitech is the firm that manufactures EmbryoScope.

### Results of pooled analysis and subgroup analysis

Summary of results are presented in Table 3

**Table 3:** Summary of findings of RCTs for the comparison between TLI and conventional methods for incubation and embryo selection in assisted reproduction.

	Outcomes	Subgroup^a^	RR (95% CI)	*N*^b^ (studies)	*I*^*2*^	Interpretation	Quality of the evidence
Oocyte-based review	Balstocyst formation		1.08 (0.94, 1.25)	1154(2)	0%	No difference	Moderate^c^
	Good quality embryo on Day 2/3		0.89 (0.72, 1.11)	720 (3)	42%	No difference	Moderate^d^
Woman-based review	Live birth		1.23(1.06, 1.44)	843(1)	N/A	TLI better	Very low^e^^,^^f^^,^^g^
	Ongoing pregnancy		1.04(0.80, 1.36)	1403(4)	59%	No difference	Very low^f^^,^^g^^,^^h^
		TLI incubation and conventional selection vs. conventional incubation and selection	0.71 (0.49, 1.03)	364 (1)	N/A	No difference	Low^i^
		TLI incubation and selection vs. conventional incubation and selection	1.21(1.06, 1.38)	1039(3)	0%	TLI better	Very low^f^^,^^g^^,^^h^
	Clinical pregnancy		1.09(1.00,1.19)	1677(5)	0%	No difference	Very low^f^^,^^g^^,^^h^
		TLI incubation and conventional selection vs. conventional incubation and selection	0.96 (0.70, 1.31)	413 (2)	0%	No difference	Low^i^
		TLI incubation and selection vs. conventional incubation and selection	1.10(0.99, 1.22)	1039(3)	0%	No difference	Very low^f^^,^^g^^,^^h^
		TLI incubation and selection vs. TLI incubation and conventional selection	1.08(0.90, 1.30)	235(1)	N/A	No difference	Low^e^
	Miscarriage		1.27(0.58, 2.80)	1403(4)	63%	No difference	Very low^f^^,^^g^^,^^h^
		TLI incubation and conventional selection vs. conventional incubation and selection	3.10 (1.10, 8.74)	364 (1)	N/A	Conventional better	Low^e^
		TLI incubation and selection vs. conventional incubation and selection	0.76(0.54, 1.07)	1039(3)	0%	No difference	Very low^f^^,^^g^^,^^h^

I2 heterogeneity, RR risk ratio, CI confidence interval

a Subgroup analysis was conducted on all outcomes when possible.

b For clinical outcomes (clinical pregnancy, ongoing pregnancy,live birth and miscarriage), N represents number of women randomized, for laboratory outcomes (good/top/optimal quality embryo on D2/3, blastocyst formation), N represents oocytes/embryos randomized.

c Downgraded because in one study the method of random allocation and blinding was not described.

d Downgraded because all included studies did not describe random allocation

e Downgraded 2 stages because evidence comes from only one study.

f Downgraded because in one study patient were able to request the intervention.

g Downgraded because one study had other potential bias.

h Downgraded because in one study paired randomization by two envelopes, researchers were awared of allocation before enrollment.

i Downgraded 2 stages because most included population comes from one study.

#### Oocyte-based review

**Fig 2:**
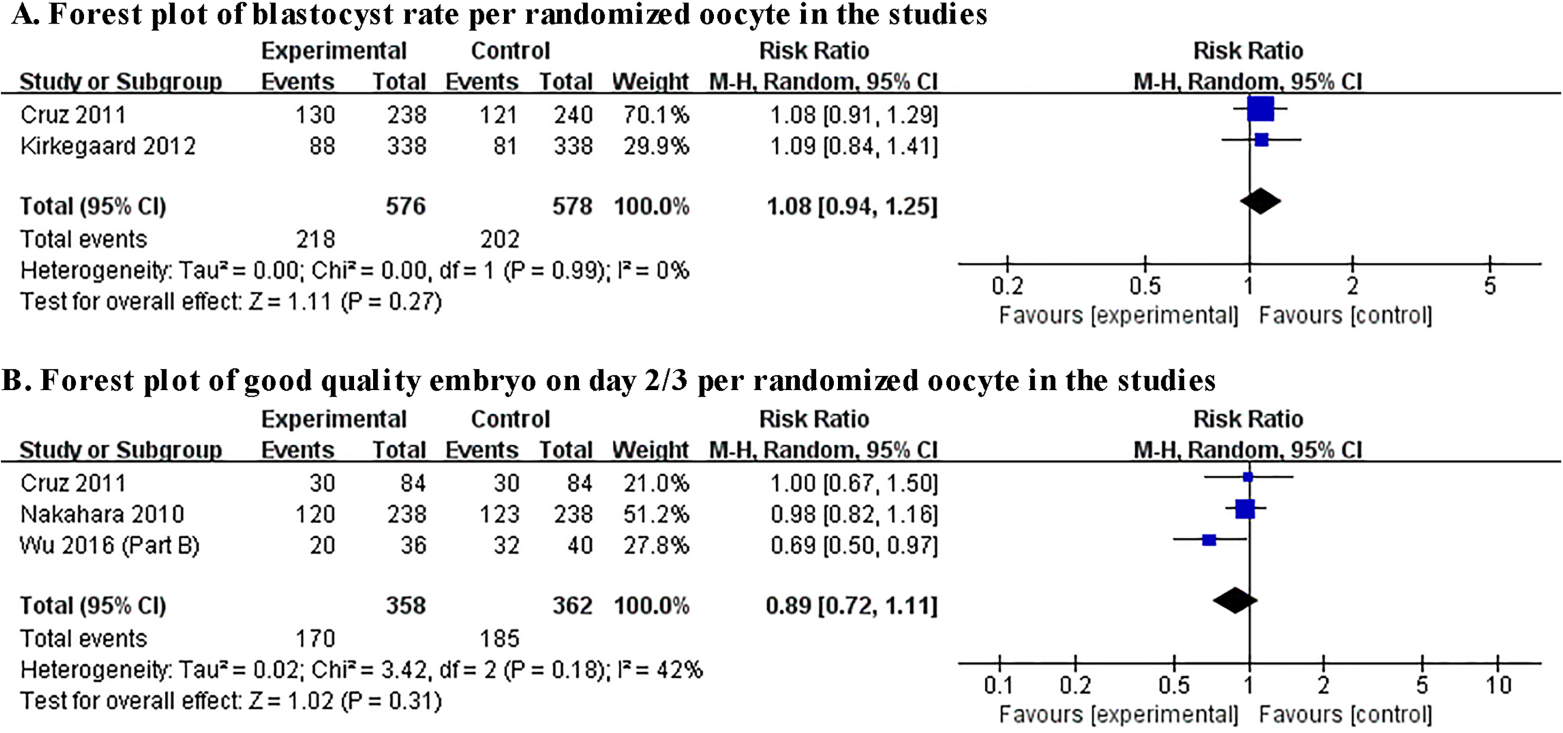
Forest plot showing the results of meta-analysis of the oocyte-based review.

Two studies presented data on blastocyst formation[24, 25], and the pooled blastocyst rate in TLI group seemed to be higher than control group, but there was no significant difference between the two groups (RR 1.08, 95% CI 0.94–1.25, I2 = 0%, two studies, including 1154 embryos) and these two studies both remove embryos out of incubator for assessment for TLI group. Three studies investigated the outcome of good quality embryo on Day 2/3[24, 26, 28], and no significant effect on this outcome was observed between TLI group and control group (RR 0.89, 95% CI 0.72–1.11, I2 = 42%, three studies, including 720 embryos). Results of pooled analysis of the oocyte-based review were showed in Fig 2.

#### Woman-based review

Live birth was only reported by one study comparing TLI incubation and embryo selection to conventional incubation and embryo selection (RR 1.23, 95%CI 1.06–1.44, I2 N/A, one study, including 843 women)[37, 39]. The pooled analysis found no significant difference between TLI group and control group for ongoing pregnancy (RR 1.04, 95% CI 0.80–1.26, I2 = 59%, four study, including 1403 women), clinical pregnancy (RR 1.09, 95% CI 1.00–1.19, I2 = 0%, five study, including 1677 women) and miscarriage (RR 1.27, 95% CI 0.58–2.80, I2 = 63%, four study, including 1403 women).

#### Subgroup analysis

Since all four studies categorizing to the oocyte-based review compared TLI incubation + conventional selection to conventional incubation and selection, subgroup analysis was only conducted for woman-based review.

Two studies reported TLI incubation and conventional selection vs. conventional incubation and selection, and no significant difference was observed between TLI incubation and conventional incubation for ongoing pregnancy rate (RR 0.73, 95% CI 0.50–1.05, I^2^ N/A, one study, including 329 women) or clinical pregnancy rate (RR 0.96, 95% CI 0.70–1.31, I^2^ = 0%, two studies, including 413 women). However, data from one study showed that miscarriage rate was significantly increased for patients randomized to the TLI group (RR 3.10, 95% CI 1.10–8.74, I^2^ N/A, one study, including 364 women)[27].

There were three studies including TLI incubation and selection vs. conventional incubation and selection, and TLI incubation and TLI selection showed significant higher ongoing pregnancy rate (RR 1.21, 95%CI 1.06–1.38, I^2^ = 0, three studies, including 1039 women) than conventional incubation + conventional selection. There were no significant differences between the two groups in clinical pregnancy rate (RR 1.10, 95%CI 0.99–1.22, I^2^ = 0, three studies, including 1039 women) and miscarriage rate (RR 0.77, 95%CI 0.55–1.07, I^2^ = 0, three studies, including 1039 women).

Only one study reported TLI incubation and selection vs. TLI incubation and conventional selection. However, the only included study did not provide data on ongoing pregnancy, live birth or miscarriage[41]. No significant difference was observed between the two groups for clinical pregnancy per randomized woman (RR 1.08, 95% CI 0.90–1.30, I^2^ N/A, one study, including 235 women).

**Fig 3:**
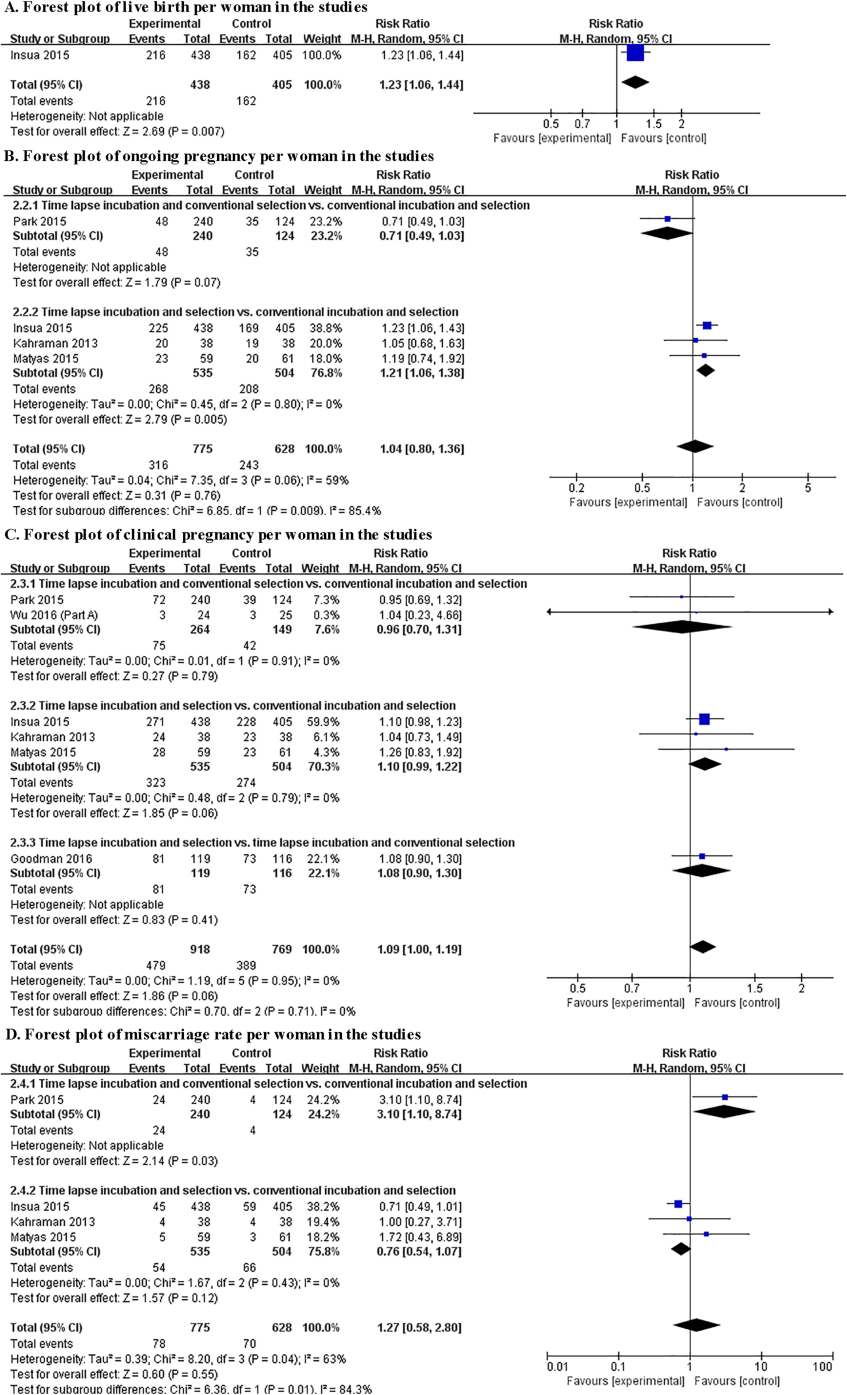
Forest plot showing the results of meta-analysis and subgroup analysis of the woman-based review.

Results of pooled analysis of the woman-based review and subgroup analysis were shown in Fig 3.

## Discussion

The present systematic review and meta-analysis shows that clinical TLI may have the potential to improve IVF outcomes but currently there is insufficient evidence to support regular TLI use when considering ongoing pregnancy rates and blastocyst formation rates. A systematic review published by Cochrane collaboration summarizing no difference between TLI and conventional methods included the same three studies[21], the literature search for Polanski’s study included the same four studies of our review[20]. Our results is mainly in line with those systematic reviews, nevertheless, much more studies and data are included in our analysis and we subgroup-analysis the included studies according to the two potential advantages of clinical TLI, making it a more robust, reliable and comprehensive quantitative assessment.

Considering the four studies that compared TLI incubation to conventional incubation and randomized oocytes, embryos incubated in TLI systems were removed out for assessment in two studies[24, 25], that eliminated the potential benefit of consistent culture and became more like the comparison between two kinds of incubators. The other two studies did not remove embryos out of TLI incubation systems during culture. However, either studies moved embryos out of TLI incubation systems or studies that did not found no significant difference in laboratory outcomes between TLI group and control group. The quality of evidence of all laboratory outcomes presented in this part is moderate, given the risk of bias in included studies.

Considering the two studies that compared TLI incubation to conventional incubation and randomized women, the pool-analysis showed no significant difference in clinical pregnancy rate between the two groups. Only one study provided ongoing pregnancy and found no significant effect of TLI incubation compared to conventional incubation on this outcome[27], but the same study observed a substantial increase in miscarriage rate in women randomized to TLI group. Although the authors explained that the increased miscarriage rate may due to the limitation of EmbryoScope for morphological monitoring because the images obtained on the monitor was not as clear and sharp as visualized in conventional inverted microscope and the focusing levels were more limited, it is still worth suspecting the potential adverse effect of TLI incubation on pregnancy. Besides, it should be highlighted that this part is heavily influenced by one study in which embryos were only cultured to day 2 and therefore there was not much difference in the amount of out of incubator handling[27]. The potential benefit of less interruption and more stable environment is more likely to be found when embryos are incubated for up to the blastocyst stage[42]. Since in this part, most available data (364/413, 88.1%) for meta-analysis of clinical pregnancy was provided by one study and ongoing pregnancy and miscarriage were only reported by single study, the quality of evidence for all outcomes is low.

Considering the studies that compared TLI incubation and embryo selection to conventional incubation and embryo selection, we observed very low quality evidence that the undisturbed culture condition and continuous embryo monitoring of clinical TLI is related with higher ongoing pregnancy rate, clinical pregnancy rate and lower miscarriage rate, but the differences of clinical pregnancy rate and miscarriage rate between the two groups were not statistically significant. Live birth was only reported by one study in a follow-up analysis and was found to be significantly higher in TLI group[37, 39]. The observed benefit on ongoing pregnancy was mainly based on the results of the biggest RCT (81.1%, 843/1039) in which most transfers were performed on day 3[37, 39], the other two small studies transferred single blastocyst for each included couple showed no significant increase in ongoing pregnancy rate in TLI group.

Only one RCT used TLI incubation for both groups and selected embryos with the addition of morphokinetic parameters to standard morphological assessment in TLI group[41]. This study was with adequate power calculation and showed a trend towards an increase in clinical pregnancy rate in TLI group, but no statistically significance.

Eight out of the ten included studies used EmbryoScope as TLI system, one study used PrimoVision and one used SANYO In vitro Live Cell Imaging Incubation System. Currently, several TLI systems are commercially available, the most widely used are EmbryoScope, Primo Vision and Eeva (Early Embryonic Viability Assessment, Progyny/Merck Serono). These systems differ in many ways including illumination, image capture and embryo culture. EmbryoScope is an incubator with integrated time-lapse imaging system using bright-field illumination, in which embryos are cultured individually in multi-well dishes. A tray holding the culture dishes is under constant movement to bring embryos one by one into the field of view of the inbuilt microscope at each image acquisition. Unlike EmbryoScope, Primo Vision (bright-field illumination) and Eeva (dark-field illumination) are microscopes that fit into standard incubators and facilitate group culture, both systems monitor all embryos at the same time without moving culture dishes, so these two systems provide more undisturbed environment than EmbryoScope concerning culture dish movement, heat and volatile organic compounds released from lubricants and electromagnetic effect. Based on the results of this review, no negative effect of EmbryoScope compared with conventional incubator is confirmed. Safety of Primo Vision and Eeva have been confirmed by some observational studies published in recent years[31–33, 43]. As to the efficiency, economical burden and embryo assessment time comparison among different types of TLI systems, scarce evidence is now available.

Compared with improved incubation, more promising benefit of TLI is the ability to combine evaluation of morphology and morphokinetic characteristics and therefore better identifying the most viable embryos for transfer. Four included studies used new algorithms based on morphology and morphokinetic parameters to select embryo for TLI group: two studies used a hierarchical classification described by Meseguer et al[25], one study clarified that the grading criteria they used was based on previously established morphokinetic parameters in their own laboratory and others’ shown to predict blastocyst formation and increased implantation, one study used composite score based on morphokinetic parameters, fragmentation and blastocyst formation but did not detailed the concrete algorithm. Recent studies demonstrated that embryo morphokinetic profile can be effected by many factors including fertilization type, patients group, culture media, oocyte source and so on[44–49]. Morphokinetic algorithms are often developed under specific clinical settings and laboratory environments and these algorithms may not be applicable in other conditions[49–52]. There is still considerable room for the improvement of TLI embryo selection. Development of morphokinetic algorithms that are specific to laboratory, patient group and treatment has been generally accepted and became a new trend in TLI use[52]. Inefficacy of TLI on clinical outcomes compared to conventional methods in some of the included studies may be due to the inappropriate morphokinetic algorithm using.

Several limitations and flaws of this review are worth mentioning. Limited data are available regarding advantages and disadvantages of clinical TLI. Live birth and neonatal outcomes were only reported by one study with severe risk of bias; data of embryo assessment time was only provided by one study and this study did not mention the TLI training status of embryologists which may seriously affect the time usage; the extra expense for TLI use and extra space need for TLI equipment was not mentioned in any of the included studies. The overall quality of evidence of all outcomes was considered to be moderate, low or very low, so inference based on these results should be made with caution. Confounding factors varied among included studies including day of transfer, number of embryos transferred, fresh or frozen embryo use, oocyte source, TLI type and especially, patient population. Eight out of 10 included studies investigated average-prognosis, good-prognosis and best-prognosis (egg donors) patients, only one small study randomized 31 poor-prognosis patients[28]. So whether TLI could benefit specific population of patients still needs to be further confirmed. In addition, four studies in the oocyte-based reivew randomized sibling oocytes and were designed to compare laboratory outcomes such as blastocyst formation; these studies have an inherent inability to provide reliable information on clinical outcomes such as live birth and ongoing pregnancy.

This analysis can help guide the clinical use of TLI. But more well designed RCTs are still required for providing sufficient evidence on the effectiveness of TLI. RCTs focusing on the effect of TLI on specific group of patients, the effectiveness comparison among different types of TLI systems, the potential disadvantages of this technology and the applicability of developed morphokinetic algorithms are currently very scarce.

## Supporting information

S1 File PRISMA checklist. PRISMA checklist.doc.
(DOC)
